# Dissociable effects of element-lifetime and stimulus-duration on local and global motion processing: An equivalent noise study

**DOI:** 10.1167/jov.26.6.8

**Published:** 2026-06-25

**Authors:** Balaje Vivekanandan, Steven C. Dakin

**Affiliations:** 1 School of Optometry and Vision Science, The University of Auckland, Auckland, Aotearoa New Zealand

**Keywords:** motion perception, local and global motion, direction discrimination, equivalent noise analysis, temporal integration

## Abstract

Human visual motion processing is thought to involve two stages: local motion processing and global pooling of local motion signals. We used an efficient equivalent noise paradigm to explore temporal aspects of motion processing, specifically manipulating the lifetime of moving elements and overall stimulus-duration, to examine (respectively) local and global processing. Participants judged whether the overall direction of a dot pattern was clockwise or anti-clockwise of a reference direction. An adaptive staircase determined a) the minimum direction offset required for reliable judgment of direction when dots moved in a similar direction and b) the maximum directional noise (range of directions) that still allowed participants to tell if a stimulus was moving either ±45° from the reference direction. The two sets of thresholds allowed us to infer both a) the precision of participants’ judgment of the direction of a single dot (i.e., local noise) and b) the number of samples they were effectively averaging (i.e., global sampling). Dots moved at 10 deg/s and we tested various dot lifetimes (33.36, 66.72, 133.4, and 266.8 ms) with a stimulus-duration of 1,000 ms, and various stimulus-durations (62, 125, 250, 500, 1,000, and 2,000 ms) with a dot lifetime of 133.4 ms. Longer element-lifetimes increased local directional precision but did not influence global integration (number of samples effectively averaged). Conversely, longer exposures did not greatly influence local precision, but increased global averaging by an average of 13% for every doubling of stimulus-duration. Thus, element-lifetime and stimulus-duration have dissociable effects on motion perception improving local and global motion processing, respectively.

## Introduction

The human visual system works continuously to detect and combine motion signals across space and time to maintain a coherent percept of movement within a dynamic visual environment (Cropper, 2001; Nakada & Murakami, 2023; Pack & Born, 2008). Such processing is necessary not only to deal with moving objects, but also to support visual scene segmentation, spatial orientation, and navigation (Braddick, Atkinson, & Wattam-Bell, 2003; Hesse & Tsao, 2023; Hupfeld, Distler, & Hoffmann, 2007; Rina et al., 2022; Schutz, Braun, & Gegenfurtner, 2011). Motion processing begins in the primary visual cortex (V1), which receives drive from (magnocellular) parasol retinal ganglion cells via the dorsal lateral geniculate nucleus (Masri, Grunert, & Martin, 2020; Merigan & Maunsell, 1993). V1 neurons respond to motion falling within a region of space known as their receptive field and drive cells in middle temporal (MT)/V5 area, which in turn drives cells in parietal areas involved in visual guidance of action. This hierarchy is known as the dorsal visual stream (Goodale & Milner, 1992; Merigan & Maunsell, 1993; Mishkin, Ungerleider, & Macko, 1983).

In general, V1 neurons are orientation selective (Hubel & Wiesel, 1968; Rust, Mante, Simoncelli, & Movshon, 2005; Schlykowa, van Dijk, & Ehrenstein, 1993) and can only signal motion direction perpendicular to their preferred orientation (Adelson & Movshon, 1982; Bradley, 2001; Pack & Born, 2001; Wuerger, Shapley, & Rubin, 1996). The confounding of a cells’ orientation and motion response is called the aperture problem for motion (Anderson & Burr, 1987; Ludge, Urbanczik, & Senn, 2014; Movshon & Newsome, 1996); To overcome the aperture problem, it is thought that a subsequent stage of motion processing occurs in MT/V5. MT neurons have large receptive fields that combine the outputs of multiple V1 cells making them suitable for computing the global direction/speed of two-dimensional (2D) patterns (Adelson & Movshon, 1982; Green & Pratte, 2022; Rodman & Albright, 1989; Stoner & Albright, 1992). One proposal as to how MT achieves this is via the intersection of constraints rule (Adelson & Movshon, 1982). Here the speed and direction of each moving one-dimensional component of a 2D image traces out a constraint line in velocity space, and MT neurons compute global motion direction—likely using a circuit involving divisive gain control (Rust et al., 2006)—as having a direction/speed falling at the intersection of these lines (Adelson & Movshon, 1982; Pack, 2001).

Natural environments (composed of texture and contours) can contain a wide range of motion signals including transparent motion, which results from the independent motion of spatially superimposed textures (Curran, Hibbard, & Johnston, 2007). In such cases, the intersection of constraints rule does not fully account for how the visual system computes motion direction (Ferrera & Wilson, 1990) and so other strategies have been proposed, for example, population vector averaging (computed over the neuronal responses of channels tuned to different directions), maximum likelihood estimation (the direction that maximizes the probability that a particular direction of motion generated the observed neuronal response) (Georgopoulos, Schwartz, & Kettner, 1986; Webb, Ledgeway, & McGraw, 2007), and feature tracking (motion of 2D features can provide unambiguous information about pattern motion) (Del Viva & Morrone, 1998; Mingolla, Ledgeway, & McGraw, 1992). Other computational models of motion processing have been developed to elucidate how local (Adelson & Bergen, 1985; Clifford & Ibbotson, 2002; Nagano, Hirahara, & Urushihara, 2004; van Santen & Sperling, 1984; van Santen & Sperling, 1985; Watson & Ahumada, 1985; Werner, 1961) and global (Baker & Bair, 2016; Deneve, Latham, & Pouget, 1999; Koechlin, Anton, & Burnod, 1999; Rust et al., 2006; Webb et al., 2007) motion systems deal with naturalistic motion. Alternatively, it has been proposed that it is possible to estimate global motion in the absence of coherent local motion signals through either attentional tracking of the position of objects or features (Allard & Arleo, 2022; Allard & Faubert, 2013; Cavanagh, 1992; Lu & Sperling, 2001). Consistent with this view, deficits in global motion perception may arise not from impaired integration of local motion signals pe se, but from impaired segregation of signal elements from noise, as demonstrated by Mansouri and Hess (2006) in amblyopic individuals.

A considerable body of evidence indicates that motion perception is impaired in various neurological conditions. A popular psychophysical paradigm in a clinical setting is to measure motion coherence thresholds (MCTs)—the minimum proportion of signal dots (moving in the same direction) intermixed with noise dots (moving randomly) required to reliably detect global direction of motion (Newsome & Pare, 1988). Poor performance on MCT tasks is observed in various neurodevelopmental (Annaz et al., 2010; Manning, Charman, & Pellicano, 2015; Milne et al., 2002; Talcott, Hansen, Assoku, & Stein, 2000), neurodegenerative (Gilmore, Wenk, Naylor, & Koss, 1994; Raz et al., 2011; Trick, Steinman, & Amyot, 1995), and acquired brain disorders (Alnawmasi et al., 2019; van der Zee, Stiers, Lagae, & Evenhuis, 2021).

Consider the widely reported deficit in motion processing in people with autism spectrum disorder (Bertone, Mottron, Jelenic, & Faubert, 2003; Del Viva, Igliozzi, Tancredi, & Brizzolara, 2006; Milne et al., 2002; Robertson, Martin, Baker, & Baron-Cohen, 2012). Although often attributed to failure of global motion processing, such deficits could arise from impairments in either local or global motion processing (Dakin & Frith, 2005). Indeed, although some studies suggest impaired global integration (Davis, Bockbrader, Murphy, Hetrick, & O'Donnell, 2006; Milne et al., 2002; Pellicano, Gibson, Maybery, Durkin, & Badcock, 2005), others ascribe it to a more general deficit in processing complex (second-order) stimuli (Bertone, Mottron, Jelenic, & Faubert, 2003), whereas yet other research reports intact global integration (Del Viva et al., 2006; Vandenbroucke, Scholte, van Engeland, Lamme, & Kemner, 2008; Zaidel, Goin-Kochel, & Angelaki, 2015), but increased inherent visual noise (Vandenbroucke et al., 2008; Zaidel et al., 2015). Similarly, although earlier findings of poor motion processing in dyslexia were ascribed to deficits in global motion integration (Cornelissen, Richardson, Mason, Fowler, & Stein, 1995; Hansen, Stein, Orde, Winter, & Talcott, 2001; Raymond & Sorensen, 1998; Talcott et al., 1998), more recent research points toward a general difficulty in processing temporal rather than motion-specific information (Conlon, Lilleskaret, Wright, & Stuksrud, 2013; Johnston, Pitchford, Roach, & Ledgeway, 2016; Manning, Hassall, et al., 2022; Scheuerpflug et al., 2004).

Given the inconsistency in findings (and their interpretation), efforts have been made to better identify the cause of motion processing deficits in various clinical conditions, for example, through the use of a limited-lifetime MCT paradigm (Baker & Hess, 1998; Baker, Hess, & Zihl, 1991; Todd & Norman, 1995). Here each member of a group of visual elements moves for a limited time before being relocated. This approach prevents observers’ tracking individual elements to promote global motion integration (Festa & Welch, 1997; Hadad, Schwartz, Maurer, & Lewis, 2015). However, because global motion integration relies on local motion detection, it is still the case that any impairment observed with a limited lifetime stimulus in the context of an MCT paradigm cannot discount the impact of local motion-processing deficits. Hence, to understand the cause of impaired motion perception, we need paradigms that directly quantify the limits of both local and global motion processing.

**Figure 1.**
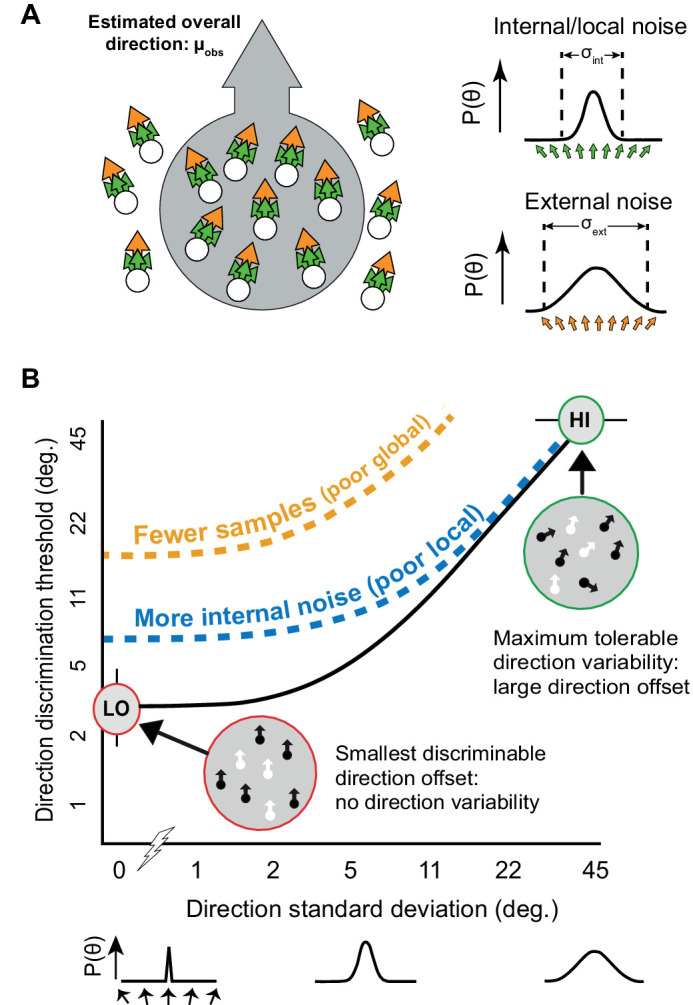
(**A**) Our ability to judge the overall/mean direction (μ_obs_) of a set of moving dots is limited by three factors. First, the range of directions present (σ_ext_); second, the precision with which we can estimate local direction (of any one dot σ_int_); and third, the number of dot directions we can effectively average (*N*_samp_; gray-shaded region). (**B**) Graph relating direction discrimination thresholds to the standard deviation of dot directions (external noise). The solid black line represents an equivalent noise (EQN) function estimated from two maximally informative thresholds: LO) the directional offset required to reliably report the direction of a set of elements moving with no directional variability and HI) the maximum tolerable directional variability for the observer to reliably report direction with a large directional cue applied (in this case, ±45°; Tibber et al., 2014). Lower sampling is represented by the dashed yellow line, which is an upward shifted EQN function. Higher internal noise is represented by the dashed blue line, which shows an initial upward shift of the EQN function at low external noise levels.

Equivalent noise (EQN) analysis (Figure 1) allows one to quantify the effects of local and global noise on performance of a perceptual task (Dakin, Mareschal, & Bex, 2005a; Dakin, Mareschal, & Bex, 2005b; Lu & Dosher, 1999; Pavan et al., 2023; Pelli & Farell, 1999). For motion integration (Figure 1a), stimuli consist of a set of dots whose directions (orange arrows) are drawn from a Gaussian distribution and where observers indicate the overall (mean) direction of the moving dots. EQN analysis assumes that the performance with a noiseless stimulus (where dots all move in the same direction)—compared with performance in a high-noise condition (where there is more variability in dot-direction)—is constrained largely by internal noise (Barlow, 1956)—the precision with which observers can estimate the direction of any one dot (the limit on local motion processing shown by the green arrows in Figure 1a). Note that although EQN studies assume averaging occurs across all noise levels, some have suggested averaging might not be occurring at low noise levels (Allard & Cavanagh, 2012). Under the assumptions of EQN analysis performance is quantified by adding more and more external noise (i.e., increasing the width of the Gaussian distribution from which true dot directions are drawn) until observers’ judgments of overall direction begin to deteriorate. The level of external noise at which this occurs is called the equivalent internal noise (σ_int_) (Dakin et al., 2005a). As external noise increases beyond this point its influence swamps the effect of internal/local noise so that performance is now largely governed by the number of dots one can effectively globally average (*N*_samp_) (Dakin et al., 2005a; Mareschal, Bex, & Dakin, 2008; Pavan et al., 2023). The two measures (σ_int_ and *N*_samp_) derived from behavioral thresholds provide detailed information about underlying mechanisms of the motion-processing system than MCT measurements alone (Dakin et al., 2005a). More formally then, if:

a. an observers’ ability to estimate the overall direction is quantified by the threshold offset (σ_obs_, the smallest offset of the overall direction that observers can reliably discriminate from some reference direction),
b. the standard deviation of directions presented within a stimulus is varied across trials (external noise, σ_ext_),

then the EQN analysis states that σ_ext_, σ_int_, and *N*_samp_ limit performance (σ_obs_) according to:

(1) $$\begin{array}{c} \;\sigma_{\mathrm{obs}}=\sqrt{\;\frac{\sigma_{\mathrm{int}}^{2}+\sigma_{\mathrm{ext}}^{2}}{N_{\mathrm{samp}}}}\; \end{array}$$

Note that, in contrast with the MCT paradigm—which uses stimuli containing a mixture of signal and noise elements—EQN stimuli are composed entirely of signal elements (whose directions are drawn from a Gaussian distribution). This design allows for a direct comparison of the observed behavior to the ideal observer to separate the impact of local and global motion processing on performance. Note, however, that EQN is a psychophysical paradigm and, as such, is concerned solely with characterizing behavior; it cannot determine how observers are actually performing the task. However, it can indicate limits and—for example, were an estimate of *N*_samp_ to exceed 1, then we can state unequivocally that observers are behaving as if they are averaging directions from multiple moving elements.

EQN typically requires estimation of thresholds at multiple levels of external noise, necessitating a large number of trials. Such an approach would not be feasible in a clinical setting, prompting the development of an efficient version of EQN analysis illustrated in Figure 1B. This technique requires estimation of thresholds only under two conditions—no noise and high noise—and allows one to rapidly quantify limits on local and global motion processing (Manning, Tibber, Charman, Dakin, & Pellicano, 2015; Tibber, Kelly, Jansari, Dakin, & Shepherd, 2014). Such an approach performs a two-parameter fit to two data points and, as such, cannot test the validity of the EQN approach (which we would argue has been established across dozen of different studies; Manning, Hulks, Tibber, & Dakin, 2022; Manning, Tibber, et al., 2015; Manning, Tibber, & Dakin, 2017; Tibber et al., 2015; Tibber et al., 2014). Alternative external noise models have been developed that use more than two parameters—such as the nonlinear perceptual template model proposed by Lu and Dosher (1998) that characterizes how parameters such as external noise, stimulus gain, internal additive noise, and internal multiplicative noise influence performance in perceptual tasks. Lu, Liu, and Dosher (2000) applied this approach to motion direction discrimination to explore how attention modulates such noise and gain parameters of the perceptual template model in motion perception. Other nonlinear external noise models with more than two parameters include the generalized noisy, inefficient observer model by (Gorea, Belkoura, & Solomon, 2014) and the ideal discriminator model by (Watamaniuk, 1993), both of which allow for a more flexible characterization of external noise and other parameters influencing performance. We briefly discuss these models in the Discussion.

EQN has been used to investigate motion perception in typically developing children (Bogfjellmo, Bex, & Falkenberg, 2014; Manning, Dakin, Tibber, & Pellicano, 2014), children with neurodevelopmental disorders (Manning, Hulks, et al., 2022; Manning, Tibber, et al., 2015; Manning et al., 2017), and in adults with amblyopia (Husk & Hess, 2013; Joshi, Simmers, & Jeon, 2016), glaucoma (Falkenberg & Bex, 2007), and migraine (Tibber et al., 2014), as well as healthy older adults (Bogfjellmo, Bex, & Falkenberg, 2013; Joshi, Simmers, & Jeon, 2021; Orchard, Dakin, & van Boxtel, 2022). EQN analysis reveals higher internal noise levels in younger, typically developing children (around 5 years old) reaching an adult-like level at around 7 years old. The effective number of samples used increases with age (Bogfjellmo et al., 2014; Manning et al., 2014). Performance of people with autism spectrum disorder indicates enhanced integration of motion signals (great sample size) without differences in local noise compared with typically developing children (Manning, Tibber, et al., 2015; Manning et al., 2017). Conversely, children with dyslexia exhibited an opposite pattern to children with autism in motion tasks, with increased internal noise and no significant differences in global sampling compared with typically developing children (Manning, Hulks, et al., 2022). These findings demonstrate that the EQN paradigm can isolate specific motion processing deficits and so potentially support differentiation of different disorders based on patterns of deficits in local and global processing.

We noted earlier that limited-lifetime MCT stimuli have been used to prevent individual dot tracking in an effort to target global motion processing. The vagaries of interpreting MCT data means that we cannot know if this works. Here we use the EQN approach to see what limiting stimulus lifetime has on local and global motion processing. In this study, we manipulated element-lifetime and stimulus-duration and used EQN to explore temporal properties of local and global processing in a direction-discrimination task.

## Methods

### Participants

Twelve participants (aged 24–54 years; 7 females) took part, all with a best-corrected visual acuity of 6/9.5 or better, which was confirmed using 5-m monocular viewing of a standard LogMAR visual acuity chart. This study was conducted in accordance with the Declaration of Helsinki guidelines and operated under an ethics protocol approved by the University of Auckland Human Participants Ethics Committee.

### Apparatus

Stimuli were displayed on a ViewSonic 27in LCD-IPS monitor operating at its native resolution of 1,920 × 1,080 pixels with a refresh rate of 240 Hz. Participants viewed the stimulus seated 75-cm from the screen. The stimulus was generated and presented under MATLAB (MathWorks, Natick, MA) using elements of the Psychtoolbox (Kleiner et al., 2007; Pelli, 1997). Observers reported the stimulus direction (clockwise/anti-clockwise) using a wireless numeric keypad.

### Stimuli

**Figure 2.**
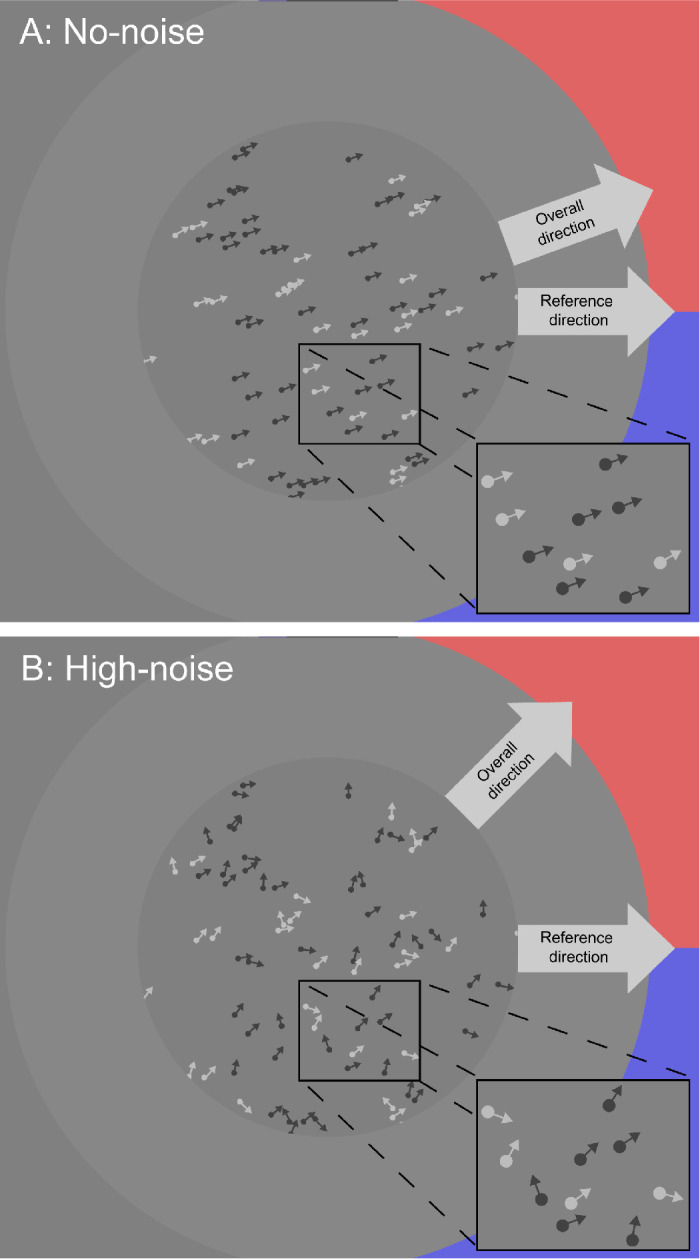
Schematic representation of the stimuli in the motion-direction discrimination task. Participants report if the overall direction (denoted by the oblique gray arrow) is clockwise (blue) or anti-clockwise (red) of the reference direction (the rightwards gray arrow). (**A**) In the no-noise condition, all elements move in the same direction (here 20° anti-clockwise from the reference), whereas in the (**B**) high-noise condition, directions are drawn from a wrapped Gaussian distribution with a mean of 45° and a standard deviation that is set by QUEST to elicit 83% correct judgment of overall direction.

Stimuli consisted of a mixture of black and white dots (diameter 0.23 deg) presented on a gray background (Figure 2). Movies subtended 40.94 deg × 25.91 deg and played at 240 Hz. At any one time, there were 128 dots displayed, with each dot moving at a speed of 10 deg/s within a circle or radius 7.78 deg, which was surrounded by a gray annulus (outer radius of 13.16 deg). Dot directions were drawn from a wrapped Gaussian distribution whose mean was either clockwise or anti-clockwise of a cardinal-reference direction (north/south/east/west, randomly selected on trial-by-trial basis; “east” in Figure 2). For each of the four cardinal directions, the stimulus display was divided into four quadrants. Red and blue panels—present throughout stimulus presentation and the response period—appeared in two quadrants adjacent to the reference direction on that trial. These colored panels indicated potential target directions, either clockwise or anti-clockwise from the reference directions. For example, if the cardinal-reference direction was east (as shown in Figure 2), the red and blue appeared on top right and bottom right, representing clockwise or anti-clockwise from the reference direction.

### Procedure

On a given trial, participants were presented with a moving dot pattern whose overall direction was offset from the reference direction by an amount referred to as the cue. After each trial, participants reported if the overall direction was either clockwise or anti-clockwise of the reference direction. Figure 2 illustrates that they did this by indicating if the pattern was moving more toward either the red or blue panels by pressing one of two keys. After they had made their response, the program advanced to the next trial.

After a practice run, the main experiment commenced. Each block of 48 trials comprised two randomly interleaved staircases/runs (each 24 trials) where the cue was under the control of QUEST (Watson & Pelli, 1983). QUEST sought to determine the cue level leading to 83% correct identification of the overall stimulus direction (clockwise or anti-clockwise of the reference direction). One staircase (no noise) presented stimuli with no direction variability (Figure 2A) and estimated the minimum direction offset required for reliable judgment of direction of a noiseless stimulus. The other staircase (high noise) (Figure 2B) used a stimulus with differing levels of directional variability where the dots’ overall direction could be either ±45° or −45° from the reference direction. A QUEST staircase estimated the maximum level of direction variability that could be tolerated for observers to correctly classify stimulus direction as either clockwise or anti-clockwise 83% of the time. These two measures are the basis of the efficient EQN paradigm (Manning, Tibber, et al., 2015; Tibber et al., 2014). The influence of trial number on the reliability of internal noise (σ_int_) and global sampling (*N*_samp_) estimates was explored using a Monte Carlo simulation, as described in the Appendix.

Using the EQN motion direction-discrimination paradigm, we examined how σ_int_ (accuracy of single-element motion direction; local motion precision) and *N*_samp_ (effective number of samples used to combine information; global motion integration) were impacted by limiting the lifetime of moving dots and by limiting the overall duration of the stimuli. The first set of conditions comprised a range of element-lifetimes (33.4, 66.7, 133.4, and 266.8 ms) while the stimulus-duration was maintained at 1,000 ms. The second set of conditions comprised a range of stimulus-durations (62, 125, 250, 500, 1,000, and 2,000 ms) while the element-lifetime was maintained at 133.4 ms. Note that the fixed 1,000-ms stimulus-duration and fixed 133.4-ms element-lifetime were selected—based on pilot observations and on the literature—to elicit good (near-asymptotic) behavior. Each participant completed a minimum of three blocks for each condition.

### EQN and data analysis

For the results of a run to be included in the analysis the following conditions had to be met: a) two or more response reversals occurred in the run and b) the estimated threshold from QUEST for that run was greater than 0. For manipulation of element-lifetime, a total of 171 runs were completed, of which 5% were excluded based on the aforementioned criteria. For stimulus-duration manipulation, 253 runs were completed; 4.5% of the runs were excluded.

In terms of data analysis, we performed a nonparametric bootstrapping procedure to the direction discrimination threshold data across valid runs. Specifically, for each element-lifetime and stimulus-duration for each participant, we generated 1,000 bootstrap samples by resampling estimated thresholds—derived from valid runs—with replacement. For example, if a participant contributed four valid runs for a given condition, each bootstrap sample consisted of four runs randomly drawn from this set, allowing the same run to be selected multiple times while others could be omitted. This approach provides an estimate of variability across runs. However, given that the number of runs was small, the resulting bootstrap distribution was limited to a smaller set of possible values and may not fully capture true underlying variability. Recall, though, that these estimates are only used to provide a graphical representation of uncertainty: the error bars. For each bootstrap sample, thresholds were averaged across runs and fitted with the EQN model to derive the estimates of local noise (σ_int_) and global sampling (*N*_samp_) using the approach mentioned above (Manning, Tibber, et al., 2015). We estimated the standard deviation of the resulting distribution of 1,000 samples of each parameter to quantify its variability. Such an estimate (given the way it was calculated) can only quantify between-run variability (because, by using QUEST estimates—rather than trial-by-trial responses—it necessarily ignores within-run uncertainty).

For each participant, a two-parameter (slope and intercept) simple linear regression model was applied to each EQN fit parameter—σ_int_ and *N*_samp_—to quantify that parameters’ relationship to both element-lifetime and stimulus-duration. We performed two sets of regressions, where when both predicted (σ_int_ and *N*_samp_) and predictor (lifetime or duration) variables were log transformed (log–log) or when only lifetime or duration were log transformed (log–linear). To choose the most appropriate model transformation, we compared the *R*^2^ values of log–log and log–linear regression fits using a paired sample *t*-test. For conditions that manipulated stimulus-duration, the log–log model explained significantly more variance for *N*_samp_ (*R*^2^ = 0.43 vs 0.40; *p* = 0.025) but not for σ_int_ (*R*^2^ = 0.18 vs 0.18; *p* = 0.949). When element-lifetime was manipulated, the difference between the models was not significant for both *N*_samp_ (*R*^2^ = 0.16 vs 0.15; *p* = 0.366) and σ_int_ (*R*^2^ = 0.37 vs 0.34; *p* = 0.064), although the log–log model explained slightly more variance for σ_int_. Overall, these results indicate that log–log transformation led to a better fit and so was used for this and subsequent analyses. Then, we tested whether the slopes of the log–log transformed regression model were significantly different from zero using a one-sample *t*-test and a sign-text (*p* < 0.05), to verify the presence of a systematic effect of element-lifetime or stimulus-duration on σ_int_ and *N*_samp_.

## Results

### Effect of varying element-lifetime on local noise and global sampling

**Figure 3.**
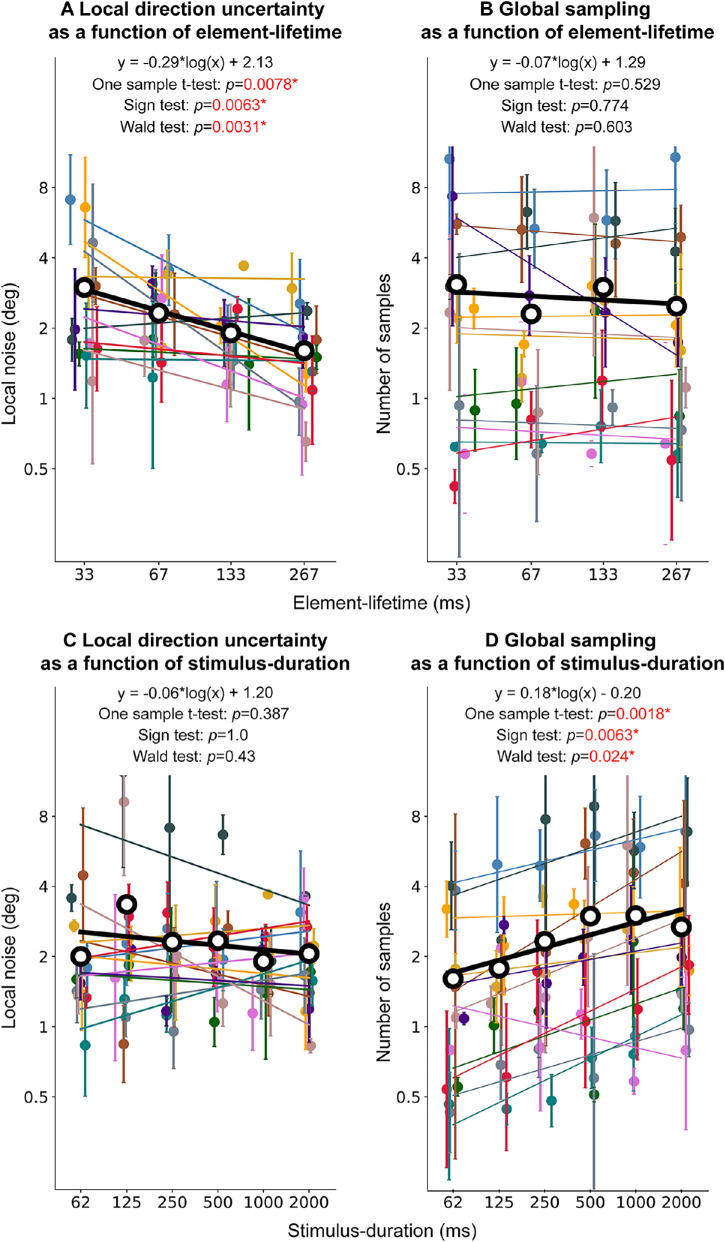
(**A**, **C**) local noise (σ_int_) and (**B,** **D**) global sampling (*N*_samp_) plot as a function of (**A**, **B**) element-lifetime and (**C**, **D**) stimulus-duration. Slope and intercept values from the linear regression on the averaged data are presented below the respective subheadings in the form of regression equations. Reported *p* values indicate whether the slopes significantly differed from 0 (*p* < 0.05). Symbol color codes participant, and large white disks represent the mean of all participants for that condition. Thicker black lines represent the linear regression model of the means. Note the significant effect of element-lifetime on (**A**) local noise but not (**B**) global sampling, and the significant effect of duration on (**D**) global sampling but not (**C**) local noise.

Figures 3A and 3B plot the estimated σ_int_ (local noise) and *N*_samp_ (global sampling) as a function of element-lifetime, respectively. For the different element-lifetime conditions, the local noise averaged across all 12 participants was: 33 ms, 3.01 ± 1.94°; 67 ms, 2.38 ± 0.91°; 133 ms, 1.91 ± 0.65°; and 267 ms, 1.61 ± 0.69°. The mean log slope and log intercept from the regression were −0.26 and 1.84. A one-sample *t*-test and a sign-test conducted on the slope values indicates significantly differing from zero (*p* = 0.0078) and (*p* = 0.0063), respectively: thus, increased lifetime reduces local noise. Averaged *N*_samp_ values for each element-lifetime condition were 33 ms, 3.101 ± 3.01; 67 ms, 2.38 ± 2.36; 133 ms, 2.98 ± 1.95; and 267 ms, 2.48 ± 2.86. The mean log slope and log intercept from the regression were −0.04 and 0.77. A one-sample *t*-test and a sign-test conducted on the slope value showed no significant effect on the effective number of samples with increasing element-lifetime (*p* = 0.529 and *p* = 0.774, respectively).

### Effect of varying stimulus-duration on local noise and global sampling

Figures 3C and 3D plot σ_int_ and *N*samp, respectively, as a function of stimulus-duration. Local noise averaged across observers for each stimulus-duration were: 62 ms, 1.99 ± 1.01°; 125 ms, 3.39 ± 3.41°; 250 ms, 2.30 ± 1.57°; 500 ms, 2.34 ± 1.39°; 1,000 ms, 1.91 ± 0.65°; and 2,000 ms, 2.06 ± 0.92°. The mean log slope and log intercept were −0.04 and 0.91 from the linear regression analysis. A one-sample *t*-test and a sign-test on the slope value revealed no significant effect of stimulus-duration on local noise (*p* = 0.387 and *p* = 1.0, respectively). The averaged *N*_samp_ values for each stimulus-duration were: 62 ms, 1.63 ± 1.26; 125 ms, 1.79 ± 1.21; 250 ms, 2.29 ± 1.91; 500 ms, 2.98 ± 2.61; 1,000 ms, 3.00 ± 1.97; and 2,000 ms, 2.69 ± 2.07. The mean log slope and log intercept were 0.18 and −0.49. The mean significantly differed from 0 (*p* = 0.0018) in a one-sample *t*-test and in a sign-test (*p* = 0.0063), indicating that the effective number of samples averaged increased with increasing stimulus-duration. The regression is consistent with a mean rate of accrual of 13% (i.e., 13% more samples averaged) with every doubling of stimulus-duration.

### Effect of varying element-lifetime and stimulus-duration on performance during no-noise and high-noise staircases

**Figure 4.**
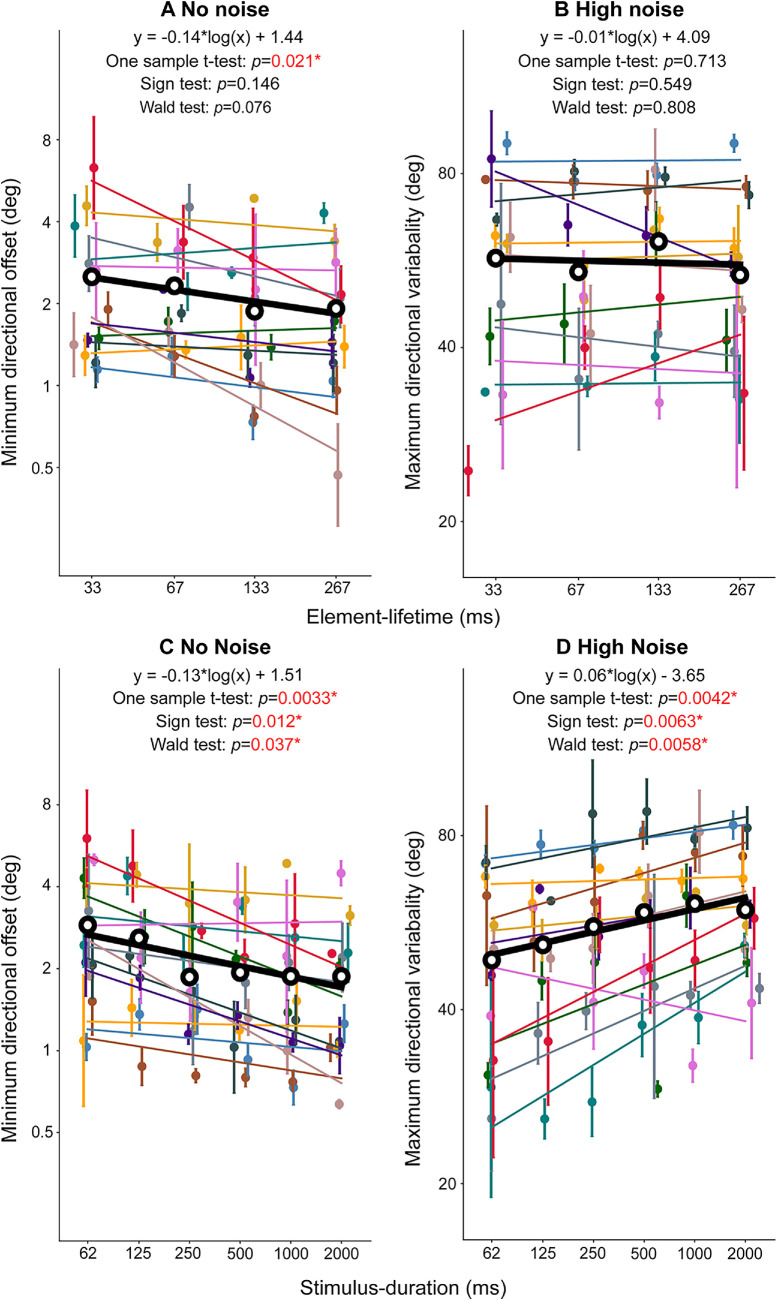
(**A, C**) Direction discrimination thresholds (degrees) from no-noise and (**B, D**) high-noise staircases plot as a function of **(A, B**) element-lifetime and (**C, D**) stimulus-duration. Slope and intercept values from the linear regression on the averaged data are presented below the respective subheadings in the form of regression equations. Reported *p* values indicate whether the slopes significantly differed from 0 (*p* < 0.05). Symbol color codes participant, and white disks represent the mean of all participants for that condition. Thicker black lines represent the linear regression model of the means.

Figure 4 plots the two raw threshold measures used to estimate local noise and effective sampling: individual estimates of minimum discriminable direction offset with no directional variability and the maximum tolerable directional variability for a ±45° directional offset discrimination. Note the similarity of trends for performance during the no-noise staircase (Figures 4A, 4C) and internal noise estimates (Figures 3A and 3C), and of performance during the high-noise staircase (Figures 4B and 4D) and estimated global sampling (Figures 3B and 3D). This is exactly in line with the predictions of an EQN approach. In no noise, performance is limited by the observers’ ability to discriminate the direction of a single element (although the models assume they average to arrive at a performance estimate) and under high noise, performance is limited by the (effective) number of samples they can average (with the effect of noise on any one sample being washed out).

**Figure 5.**
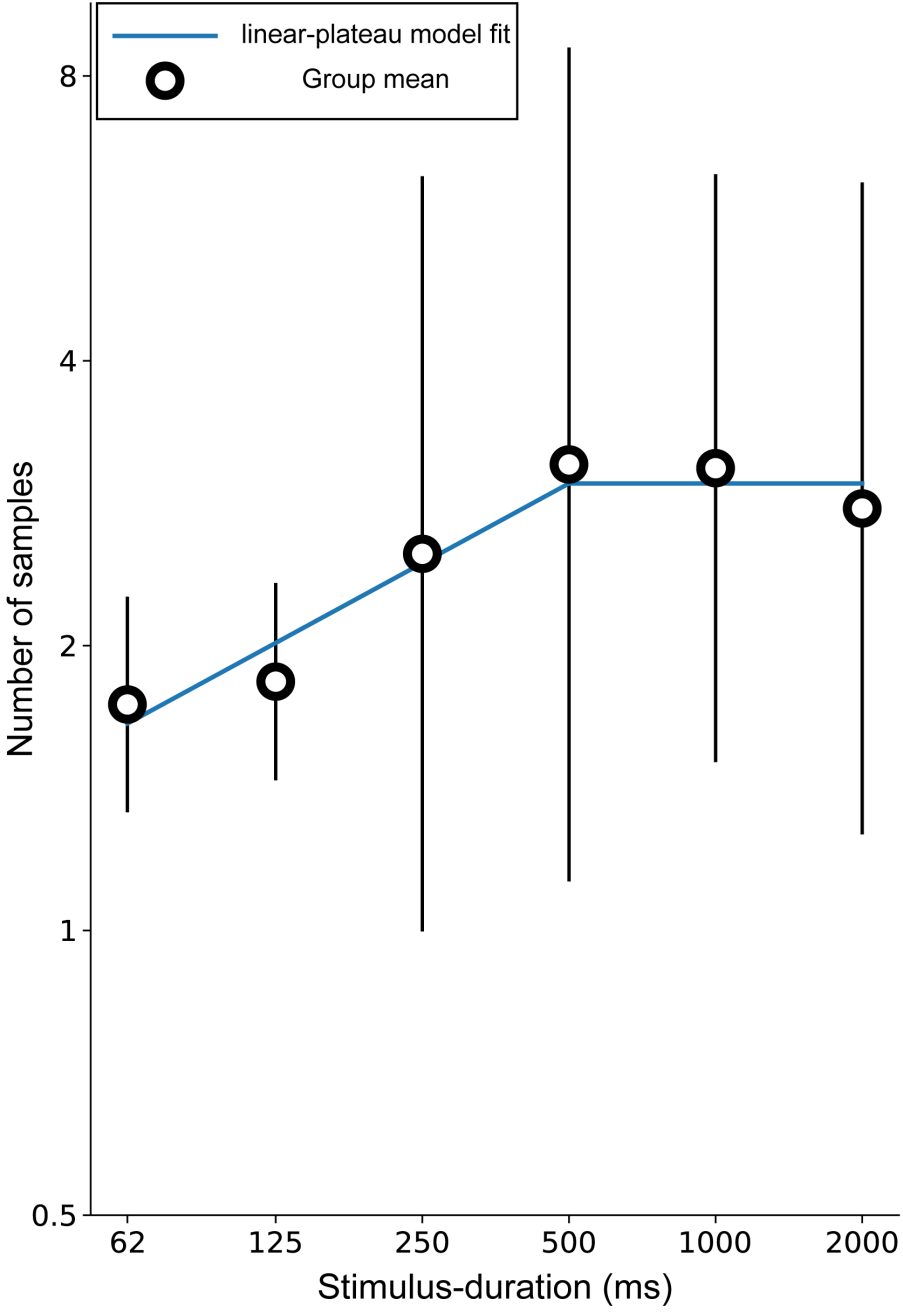
Mean global sampling estimates (*N*_samp_) plotted as a function of stimulus-duration and fit with the linear-plateau model. Group mean estimates for each stimulus-duration is represented by the white discs. Error bars represent the between-observer standard deviation of *N*_samp_ at each stimulus-duration. The solid blue line shows the best fitting linear-plateau model applied to the log-transformed *N*_samp_ values. Data are plotted on a log scale for both axes. The model captures an increase in *N*_samp_ with stimulus-duration followed by a plateau (of 3.0 samples) beyond the estimated breakpoint (500 ms), indicating that additional stimulus-duration does not lead to increase in effective global sampling.

## Discussion

To summarize, decreasing element-lifetime (for a given stimulus-duration) degrades direction discrimination in a manner that is consistent with a systematic increase in local noise, but not with a change in global sampling. Conversely, decreasing stimulus-duration (while keeping element-lifetime constant) reduces the effective global number of samples used to judge motion direction while having an insignificant effect on local noise.

Previous work has shown that limiting the lifetime of stimulus elements elevates the amount of motion coherence required to judge direction (Festa & Welch, 1997; Jackson et al., 2013), but why might limiting element-lifetime selectively impact local noise? First, limiting element-lifetime reduces the portion of the temporal integration period of a local motion detector that is occupied by the stimulus, so reducing that detector's response (Watamaniuk, Flinn, & Stohr, 2003). Second, reducing lifetime increases correspondence noise (the probability that a moving element will be mismatched to another element across time), leading to the activation of detectors tuned to the wrong direction (Festa & Welch, 1997; Lee & Lu, 2010). In the limit, such elevation of correspondence noise, can cause two-frame motion to appear as discrete flashes rather than motion. Collectively, reduced activation of sensors tuned to direction close to the true overall direction, and increased activation of sensors tuned away from the true overall direction, will serve to increase uncertainty about the direction of single elements (i.e., local noise).

We also report that decreasing the stimulus-duration reduces the effective number of samples used to judge motion direction while having an insignificant effect on local noise. Again, previous studies have reported that shorter stimulus-durations worsen performance in motion integration tasks (Aaen-Stockdale, Ledgeway, & Hess, 2007; Burr & Santoro, 2001; Caudek, Domini, & Di Luca, 2002; Hill & Raymond, 2002; Robertson et al., 2012). It has been proposed that such deficits simply arise from the visual system being able to collect less information about the stimulus. Previous studies have reported variable temporal integration limits (the duration beyond which performance plateaus) for coherence thresholds measured with random dot patterns, such as 440 ms in Williams and Sekuler (1984), 580 ms in Watamaniuk, Sekuler, and Williams (1989), 465 ms in Watamaniuk and Sekuler (1992), 1,000 ms in Katz, Hennig, Cormack, and Huk (2015), and up to 3,000 ms in Burr and Santoro (2001). We considered whether our observers’ effective global sampling reached an asymptotic limit with stimulus-duration. To this end, we applied a linear-plateau model to the log-transformed global sampling estimates as a function of log-transformed stimulus-duration for each participant, as well as to the group mean (Figure 5). The linear-plateau model assumes that global sampling increases logarithmically with stimulus-duration up to a breakpoint, after which it remains constant. Specifically, the model is defined by three parameters: an intercept *a*, and a slope *b*, which describes the rate of increase prior to the breakpoint (*t*_0_), marking the onset of the plateau. For stimulus-durations shorter than *t*_0_, the model predicts a linear increase (*a* + *b*.log(*t*)), where *t* denotes the stimulus-duration. For durations longer than *t*_0_, the response is constrained to a constant value equal to the linear prediction at the breakpoint (*a* + *b*.log(*t*_0_)), resulting in a plateau.

We compared the three-parameter linear-plateau model against the two-parameter linear regression model, which we used to quantify the relationship between EQN fit parameters and element-lifetime or stimulus-duration, using the Akaike Information criterion (AIC). The AIC quantifies model quality by balancing how well a model captures the observed data with how many parameters it uses to do so. It combines a likelihood-based measure of fit with an adjustment that favors models that achieve good explanatory power with fewer assumptions. This provides an objective basis for deciding whether the linear-plateau model offers a statistically better account of the data compared with simple linear regression. The linear-plateau model provided a superior fit for only 1 of the 12 individual participants, but offered a better account of the group mean data when compared with simple linear-regression model. For the mean data, the linear-plateau model explained 94% of the variance (*R*^2^ = 0.94) by placing the breakpoint at 500 ms (Figure 5). Prior to the breakpoint, effective global sampling estimates increased significantly with stimulus-duration (slope, 0.28; intercept, −0.65; *p* = 0.01). The breakpoint itself was significant (*p* < 0.001) consistent with a plateau beyond 500 ms. Consistent with this, the AIC values for the linear regression model and linear-plateau model are −21.66 and −28.59, respectively, with the lower AIC value for the latter model indicating a better fit.

Watamaniuk (1993) investigated the effect of manipulating stimulus parameters such as duration, density, and area on observers’ performance in a motion direction discrimination task. He first introduced an ideal-discriminator model (IDM) to evaluate the efficiency of the visual system and to identify factors that differentiate human performance from an ideal observer. With the IDM, he found that performance decreased proportionally to the square root of duration and did not hit a temporal integration limit. He also observed that the IDM always outperformed human observers’ performance and so he developed a local–global noise model to make the IDM mimic a human observer. This model incorporated multiple parameters, such as spatial and temporal integration limits, a scaling factor to account for loss of information due to stimulus construction and internal noise. Watamaniuk's own local–global noise model provided a closer fit to the human observers’ data and successfully captured key trends in performance, offering a more accurate representation of human motion perception behavior.

Outside of motion processing, our results accord with observations made by Gorea et al. (2014), where the authors reported that a shorter stimulus-duration reduced the effective sample size in a mean-size discrimination task. To explore whether the performance improvements over time reflects increased sampling or increased precision, the authors developed a generalized, noisy, inefficient observer model, related to the EQN. This model incorporated parameters, such as number of visible elements, number of times an observer forms an independent estimate, number of effective samples used in forming an estimate, and variance of early and late noise. Their results showed first that the thresholds for the size discrimination were lower when eight elements were presented compared with when a single element was presented, indicating a benefit from averaging across multiple elements. Second, observers used fewer samples and were decreasingly precise as the exposure duration of multiple elements decreased.

Previous clinical studies that reported impaired global motion perception have used stimulus-durations that fall in the range used here. For example, Robertson et al. (2012) examined coherent motion perception in observers with and without autism using viewing durations of 200, 400, and 1,5000 ms, which lie within the range we used (62–2,000 ms). The authors reported that group of observers with autism were less accurate than controls at shorter stimulus-duration (200 ms), which is consistent with the idea of temporal constraints affecting the performance. Sutherland and Crewther (2010) reported elevated MCTs in individuals with autism when the dot lifetime was short (100 ms) compared with an infinite dot lifetime. In addition, effects of temporal constraints have also been demonstrated in groups of people with dyslexia, where Conlon et al. (2013) reported worse performance in a motion coherence task for shorter duration (84 ms) compared with longer duration (133 ms). The temporal manipulation used in the above-mentioned studies spans a similar range of temporal conditions used in our experiments, which suggests that the larger deficits reported in clinical populations cannot be attributed solely to methodological differences in temporal parameters. Instead, they likely reflect genuine differences in motion-processing mechanisms in these clinical groups.

We cannot quantitatively map our results directly onto previous work (e.g., to say that poor performance with limited lifetime stimuli in people with dyslexia reflects not poor integration but elevated local uncertainty) because a) we do not have a clinical test group and b) prior work has largely used MCTs (for which we do not have an ideal observer model). With that said, consider the finding that children with dyslexia exhibit a ∼29% elevation in internal noise for direction discrimination (compared with controls; Manning, Hulks, et al., 2022) and that their MCTs can be elevated by more than 60% for limited lifetime stimuli (Talcott et al., 1998). We suggest that the reason for these large effect sizes with MCT stimuli (Benassi, Simonelli, Giovagnoli, & Bolzani, 2010) is that the limited lifetime of the stimulus provides a baseline/pedestal level of local noise. Control observers (with their lower internal noise) can tolerate this noise pedestal with little effect on performance, but for observers with dyslexia, their own higher levels of internal noises combines with the pedestal to have more drastic effects on performance. In this way, the modest effects we report for (say) local/internal noise could translate into larger effects with previous clinical paradigms. Given another limitation of our study—that we did not explore the interaction of element-lifetime and stimulus-duration—it is also possible that such differences are magnified with the particular combinations of parameters used.

Taken together, the results of our study suggest that manipulating element-lifetime and stimulus-duration play a role in accurately perceiving motion direction by improving precision of determining local element direction and by allowing for more information accumulation, respectively.

## Conclusions

Our findings contribute to a body of evidence demonstrating that the manipulation of element-lifetime and stimulus-duration can exert distinct effects on local and global processing of motion. Decreasing element-lifetime degrades the performance in a direction discrimination task, primarily due to the diminished accuracy of judging the direction of individual elements as a result of elevated internal noise, rather than deficits in global integration. In contrast, reducing the stimulus-duration compromises the performance in the same task by limiting our visual system to accumulate necessary information for the optimal assessment of global motion direction. By using an EQN analysis, we were able to dissociate the effects of manipulating temporal aspects of the stimulus on our local and global motion processing. This emphasizes that methods like the EQN model should be widely used to understand the motion-processing system in detail and will be particularly valuable in identifying the specific cause of motion-processing deficits that manifest in various clinical conditions.

## Figures and Tables

**Figure A1.**
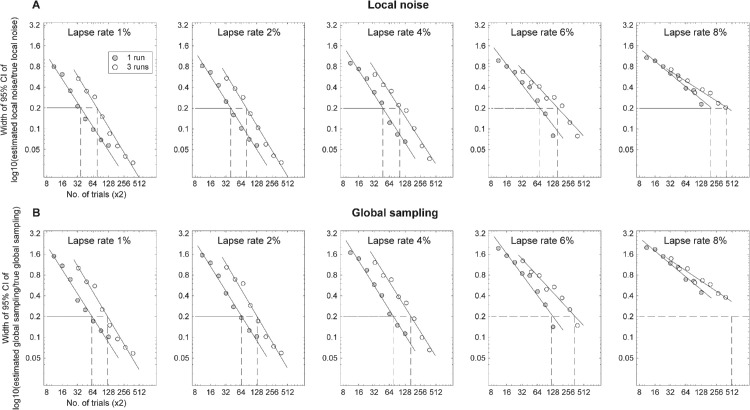
Monte Carlo simulation of trial requirements for reliable equivalent noise (EQN) estimates. (**A**) Plot of 95% confidence intervals (CIs) of the log10 ratio of simulated to true estimates across a range of trial numbers for internal noise (σ_int_), and (**B**) for global sampling (*N*_samp_). Each column depicts the CI width of various trial numbers for a given lapse rate. The solid horizontal line represents a ±0.1 CI of log_10_ ratio of simulated values to true values, and the dashed vertical line marks the minimum trial count to achieve this level of precision. Filled circles represent a single run of the indicated trial number, and the unfilled circles are for three runs totaling the same trial count. The *x*-axis label “No. of trials” denotes the number of trials per interleaved QUEST staircase (there were two: no noise and high noise) used to estimate σ_int_ and *N*_samp_.

**Figure A2.**
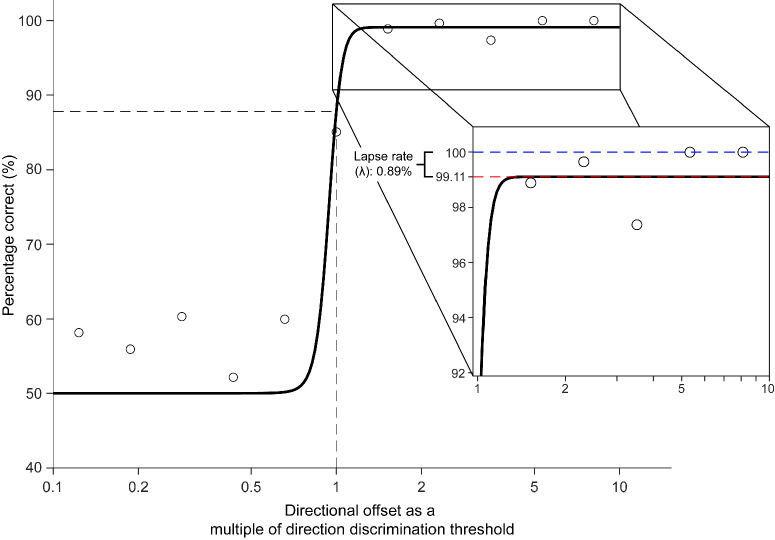
Psychometric function for the thresholds from no-noise condition pooled across all stimulus-durations and observers. The main panel shows proportion correct (%) as a function of normalized stimulus intensity (directional offset expressed as a multiple of direction discrimination threshold) on a log-scaled *x*-axis. Open circles represent the observed data, and the solid black curve shows the best-fitting three-parameter logistic psychometric function with the guess rate fixed at 50% for a two-alternative forced choice task. The dashed line indicates the corresponding percentage correct (87.8%) at the threshold, derived from the fitted function on pooled, normalized data. The inset shows the high-stimulus intensity region on an expanded scale, illustrating the lapse rate. The blue dashed line indicates the 100% performance, and the red dashed line indicates the upper asymptote of the fitted function (1 − λ = 99.11%). The bracket shows the difference between the two, corresponding with the estimated lapse rate of 0.89%, reflecting the small proportion of stimulus-independent errors attributable to attentional lapses or motor mistakes shared across observers.

## Appendix Appendix

We performed a Monte Carlo simulation to estimate the number of trials required to obtain reliable internal noise and global sampling estimates using our paradigm. Specifically, we simulated a two-alternative forced choice motion direction discrimination task for the no-noise and high-noise conditions using QUEST with a target performance of 83%. The initial parameters for QUEST were fixed while we co-varied the lapse rate (the probability of making a random response; 1%, 2%, 4%, 6%, and 8%) and number of trials (11, 16, 23, 32, 45, 64, 91, and 128). For each combination of lapse rate and number of trials, we ran 1,024 replications of the simulated experiments to power a bootstrap that yielded distributions of σ_int_ and *N*_samp_ for that condition. The simulated observer was also implemented using QUEST. We used the group mean and standard deviations of the no-noise and high-noise conditions. For each bootstrap, the observer's base thresholds were sampled from standard-normal distributions defined by the population means and between-observer standard deviations.

For each trial within a simulated experiment, test intensity was generated using QUEST, a response (correct/incorrect) was generated using the QUEST-simulated psychometric function with a given lapse rate, and the QUEST posterior was then updated. This was done independently for the no-noise and high-noise conditions, and the results were paired up within each replication (to mirror our study's experimental design) to estimate σ_int_ and *N*_samp_.

We did this for two experimental designs: a single run of a given trial number and a combination of three independent runs with the same total trial count as the single run, each containing two interleaved QUEST staircases.

After each simulated run, the final thresholds were obtained from QUEST posterior probability density function means and were converted to estimates of local noise (σ_int_) and effective global sampling (*N*_samp_) using the same transformation applied to the human data in our study. For the multi-run condition, estimates were combined using the geometric mean across runs.

To quantify the reliability of estimated local noise and effective global sampling (compared with the known ground-truth values), we calculated the log_10_ ratio of the simulated to true value across bootstraps and then identified the 95% confidence interval of the distribution. We did this for each combination of a given trial number and lapse rate. These confidence intervals capture the expected variability of the estimate relative to the true value with narrower intervals indicating precise estimates. In Figures A1A and A1B, we plot the 95% confidence intervals of the log_10_ ratio for σ_int_ (Figure A1A) and *N*_samp_ (Figure A1B). The solid horizontal line at 0.2 on the *y*-axis represents the ±0.1 confidence interval, and the dashed vertical line indicates the minimum number of trials required to achieve this level of precision. This minimum trial number is determined by identifying where the confidence interval width first intersects at the ±0.1 point, directly linking the simulation-derived variability to a practical recommendation for trial count.

For both equivalent noise (EQN) estimates, our results indicate that, for lapse rates lower than 6%, performing a single run with a greater number of trials yields more reliable estimates than combining multiple shorter runs with the same total trial count. This likely occurs because QUEST is an adaptive procedure that gradually refines its estimate of threshold based on accumulated responses. In a single long run, later trials are concentrated near the observer's true threshold and provide highly informative data. In contrast, splitting trials across multiple shorter runs repeatedly resets QUEST, increasing the proportion of trials spent in the early and less informative phase of the staircase. When lapse rates are low, responses are largely stimulus driven, allowing QUEST to refine the threshold estimate efficiently without interruption during a single long run. This advantage of single long run is not observed at higher lapse rates, where estimates from a single and multiple runs almost overlap, an effect that is slightly more pronounced for global sampling estimates. At higher lapse rates, a greater proportion of random response errors reduces the reliability of stimulus-driven information diminishing the benefit of continued adaptive refinement by QUEST. As a result, single long runs and multiple shorter runs of the same total trial count yield EQN estimates at higher lapse rates.

The lapse rate was estimated by using the response data from the no-noise condition—pooled across all observers and stimulus-durations—and fitting a psychometric function with an explicit lapse parameter (Figure A2). The no-noise condition was chosen because, in this condition, performance could reach 100%. This ensures that lapses primarily arise from stimulus-independent factors, such as attentional lapses or motor errors, rather than limitations imposed by external noise. To pool the data, we first normalized—on an observer-by-observer basis—the stimulus intensity by the corresponding threshold estimate obtained from the staircase. This normalization allowed responses from different observers and conditions to be combined on a common scale. Responses (correct or incorrect) as a function of a single independent variable (directional offset expressed as a multiple of individual direction discrimination threshold) were combined across all participants. Next, a three-parameter (α, threshold; β, slope; λ, lapse rate) logistic psychometric function, with a fixed guess rate of 0.5, was fit to the pooled binary data. Parameters were estimated by minimizing the negative log-likelihood of the observed binary trial responses (correct or incorrect), where the probability of a correct response at each stimulus level was given by the psychometric function. This was done across all observers/trials in the no-noise condition using the fminsearch function in MATLAB. Thus, the resulting lapse rate was computed from a full distribution of responses across stimulus levels and reflects a global estimate of the probability of attentional, or motor errors shared across observers. Figure A2 shows the fitted psychometric function resulting in a lapse rate of 0.89%.

Because our computed lapse rate is close to 1%, consider the leftmost two graphs in Figure A1. For reliable internal noise estimate in our observers, a single run of 36 trials or a combination of 3 runs adding up to 76 trials should be used. The latter approach aligns closely with our implemented protocol of 3 runs of 24 trials for each staircase—adding up to a total of 72 trials per staircase. For global sampling, given the same lapse rate, reliable estimates would require either a single run of 60 trials or 3 runs totaling 125 trials.
